# Portuguese Local Pig Breeds: Genotype Effects on Meat and Fat Quality Traits

**DOI:** 10.3390/ani10050905

**Published:** 2020-05-22

**Authors:** José Manuel Martins, Rita Fialho, André Albuquerque, José Neves, Amadeu Freitas, José Tirapicos Nunes, Rui Charneca

**Affiliations:** 1 MED—Mediterranean Institute for Agriculture, Environment and Development & Departamento de Zootecnia, Escola de Ciências e Tecnologia, Universidade de Évora, 7006-554 Évora, Portugal; jneves@uevora.pt (J.N.); aagbf@uevora.pt (A.F.); 2 MED—Mediterranean Institute for Agriculture, Environment and Development, Universidade de Évora, 7006-554 Évora, Portugal; a.ritafialho@gmail.com (R.F.); andrealb@uevora.pt (A.A.); 3 MED—Mediterranean Institute for Agriculture, Environment and Development & Departamento de Medicina Veterinária, Escola de Ciências e Tecnologia, Universidade de Évora, 7006-554 Évora, Portugal; jnunes@uevora.pt (J.T.N.); rmcc@uevora.pt (R.C.)

**Keywords:** swine, Alentejano, Bísaro, Ribatejano, meat quality, dorsal subcutaneous fat

## Abstract

**Simple Summary:**

Local breeds are generally associated with slower growth rates, higher slaughter weights, and fatter carcasses due to genetic and rearing system characteristics. When compared to intensive pig production systems, those based on European local breeds generally provide a more favourable response to the required increase in the production of high-quality pork and pork products in sustainable chains, meeting consumer demands. Reducing costs and improving the economic viability of production systems while preserving the quality of the products obtained is of vital importance. In this work, we propose that Portuguese local pig production chains could improve their performance and productivity without compromising the quality of the final product by crossing local breeds instead of crossing with modern breeds. This could help to maintain or increase local breed populations, supporting conservation of animal biodiversity.

**Abstract:**

This work investigated the contribution of cross-breeding between two local Portuguese pig breeds to the conservation of animal biodiversity and income of local pig producers. Quality traits of semimembranosus (SM), gluteus medius (GM) and dorsal subcutaneous fat (DSF) were studied in Alentejano (AL), Bísaro (BI), AL × BI, and BI × AL (Ribatejano—RI) castrated male pigs. Pigs were reared outdoors, fed ad libitum, and slaughtered at ~65 (trial 1) and 150 kg BW (trial 2). In trial 1, AL pigs showed higher SM intramuscular fat, lower total collagen, and higher soluble collagen than BI pigs, while AL × BI and BI × AL pigs showed intermediate (NS) values. AL, AL × BI, and BI × AL pigs showed higher SM myoglobin content, and AL a more intense red colour than BI pigs. Finally, AL, AL × BI, and BI × AL showed higher total lipids in DSF than BI pigs. In trial 2, SM and DSF results were similar to those obtained in trial 1. In GM, AL and BI × AL showed higher intramuscular fat than BI and AL × BI pigs, while AL, AL × BI and BI × AL showed lower total collagen content than BI pigs. In conclusion, these results suggest that RI crosses are a productive alternative, with overall muscle and DSF traits statistically not different between AL × BI and BI × AL, and similar to those observed in AL pigs.

## 1. Introduction

The increasing demand for pork products is leading to an international effort to save traditional pig breeds and develop new breeds [1]. In Portugal, the main local pig breeds are the Alentejano (AL), an Iberian type breed [2] with an extreme genetic closeness to the Iberian pig [3], and the Bísaro (BI) pig, from the Celtic type [2]. The AL pig is characterised by a low growth rate (except under “montanheira” regime) and precociously high adipogenic activity [4]. The BI pig presents a poor growth (although higher than AL), little backfat (although higher than that of industrial genotypes), and a high proportion of skin and bone [5]. These environmentally well-adapted local breeds are less productive than modern improved genotypes, and their production chains depend mainly on the marketing of meat, and fermented and dry-cured meat products with highly valued sensory characteristics [5,6,7]. Almost extinct in the 1980s [5,8], these two breeds and their traditional systems have recovered since the 1990s [6], driven by consumer concerns about animal welfare, sustainable production, and meat and meat product quality issues. Although still threatened (AL) and rare (BI) breeds [9], they currently have a high ecological, economic and social importance in their production regions [6,10,11]. Increased yields and reduced costs on these productive systems is a continuous challenge, and crossbreeding is one way of achieving it.

AL and BI breeds homelands, in the South and North of Portugal respectively [8], have contact zones in the Tagus River region. In these contact zones (e.g., Ribatejo region), crosses between the two breeds were common until the 1950s [12] and the meat and meat products obtained were highly appreciated. However, there are no data available regarding these crosses, popularly called Ribatejano (RI) pigs. This study, included in the TREASURE project dedicated to European local pig breeds, was the first to collect and analyse carcass, pork and fat data from crossbred RI pigs, using AL and BI pure animals as controls. It represents a potential new management strategy for these breeds’ production chains, while attending to societal demands for environment preservation, sustainable local agro-economy, as well as to consumers demands for quality and healthiness on regional pork products. The recovery and commercial use of these crosses could also help to preserve the pure breed populations, maintaining animal biodiversity, essential for an efficient and sustainable world food production and to meet the different needs of modern human societies [13]. In fact, loss of biodiversity may lead to an impaired response to changing environments.

Following a previous work where growth, carcass traits and loin data were presented [14], this study evaluates meat and fat quality from AL, BI, and AL × BI and BI × AL (RI crosses) pigs, reared outdoors, fed ad libitum, and slaughtered at ~65 and 150 kg BW. Physicochemical traits of semimembranosus (SM), gluteus medius (GM), and dorsal subcutaneous fat (DSF) were determined.

## 2. Materials and Methods

### 2.1. Animals and Experimental Design

Experimental procedures and animal care were performed in compliance with the ethical guidelines and regulations of the Portuguese Animal Nutrition and Welfare Commission (DGAV—Directorate-General for Food and Veterinary, Portugal), following the 2010/63/EU Directive.

Composed by two sequential trials, this work had four experimental groups, with pure Alentejano (AL) and Bísaro (BI) pigs and their reciprocal crosses, AL × BI and BI × AL (Ribatejano (RI) pigs, the common name of these crossbred). Male pigs (n = 20 for each of the 4 genotypes) surgically castrated within the 1st week of age were reared outdoors from 28.6 ± 0.5 kg BW (mean ± SEM) until ~65 kg (trial 1) and from 65.2 ± 0.4 kg to ~150 kg (trial 2). In trial 1, pigs were group-fed with commercial diets (15.5–16.6 g/100 g crude protein, 12.4–12.7 g/100 g NDF, 4.5–5 g/100 g total lipids and 14.1–14.3 MJ/kg DE—Supplementary Table S1) at estimated ad libitum consumption [15], in a single daily meal (09:00 h). In trial 2, pigs were individually fed with commercial diets (15.4–16.6 g/100 g crude protein, 12.4–12.9 g/100 g NDF, 4.7–5 g/100 g total lipids and 14.1–14.3 MJ/kg DE—Table S1) and diet refusals were measured daily. All animals had free access to water and were weighed every fortnight.

Temperature and relative humidity data were registered throughout the experimental work. Average temperature, average minimal and maximal temperature, and average relative humidity were, respectively, 11.2, 6.5, 17.4 °C, and 75.0% in trial 1 (January–April), and 21.9, 13.7, 31.1 °C, and 56.3% in trial 2 (April–October).

Slaughtered at a commercial slaughterhouse in three batches per slaughter weight, animals had free access to water but were fasted for ~16 h during lairage. Ten animals from each genotype were slaughtered at the end of trial 1 (average BW of 64.2 ± 0.3 kg) and nine animals at the end of trial 2 (average of 150.6 ± 0.9 kg BW) by exsanguination following CO_2_ stunning. Commercially reared local pigs are slaughtered at lighter weights for consumption as fresh meat or roasted pigs, or at heavier weights for the production of high quality traditional fermented and cured products [6,16] with PDO or PGI European certification.

### 2.2. Muscle and Adipose Tissue Sampling

Samples of SM muscle and of DSF were obtained from the left half carcasses at ~65 and 150 kg BW, while samples of GM muscle were collected only at ~150 kg. Samples were vacuum packaged and frozen (−20 °C) until analysis.

### 2.3. Muscles and Adipose Tissue Analyses

Leg (SM and GM) muscles represent a cut of greatest economic value and mass. Muscles ultimate pH (pH_u_) was determined 24 h post-mortem by a pH-meter with a puncture electrode (LoT406-M6-DXK-S7/25, Mettler-Toledo GmbH, Germany).

Moisture was determined in muscles and DSF according to ISO-1442 [17]. Total nitrogen from muscle samples was determined in a Leco FP-528 (Leco Corp., St. Joseph, MI, USA) by the Dumas combustion method (method 992.15) and from DSF samples by the Kjeldahl method (method 928.08) [18]. Total protein content was estimated as total nitrogen × 6.25. Total lipids were determined in muscles according to Folch, et al. [19] and in DSF by Soxhlet extraction (method 991.36) [18]. Muscles myoglobin and total collagen were determined as previously described [20] and soluble collagen according to Hill [21].

Surface colour measurements [22] of raw SM and GM samples were recorded with a CR-400 colorimeter (Konica Minolta Sensing Europe B.V., Nieuwegein, Netherlands) with a D65 illuminant, after blooming for 30 min. Individual CIE (Commission Internationale de l’Éclairage) L* (lightness), a* (redness) and b* (yellowness) values were averaged out of six random readings across muscle surface. The same procedure (without blooming) was applied to DSF samples. Chroma ($C^{*}=\surd \left(a^{*}{}^{2}+b^{*}{}^{2}\right)$, hue angle $\left(H{}^{\circ}=\;tan^{-1}\left(b^{*}/a^{*}\right)\right)$, and saturation $\left(C^{*}/L^{*}\right)$ were calculated.

### 2.4. Statistical Analysis

All data were tested for normality by the Shapiro-Wilk test. Results are presented as mean ± rSD. Data were analysed by one-way analysis of variance (ANOVA) with SPSS Statistics software (IBM SPSS Statistics for Windows, v24.0, IBM Corp., Armonk, NY, USA). Mean differences were considered statistically significant when *p* < 0.05, and *p* values between 0.05 and 0.10 were considered trends.

## 3. Results

### 3.1. Trial 1: Pigs Slaughtered at ~65 kg BW

Pigs were slaughtered at an average age of 186.2 ± 2.9 days.

#### 3.1.1. Muscle Tissue Analyses

SM physico-chemical data were affected by genotype (Table 1). Moisture was lower (*p* < 0.05) in AL and AL × BI than in BI pigs, while total intramuscular fat (IMF) was higher (*p* < 0.05) in AL than in BI pigs. Myoglobin content was higher (*p* < 0.01) in AL pigs and Ribatejano (RI) crosses than in BI pigs, whereas total collagen was lower (*p* < 0.05) in AL than in BI pigs, with their crosses showing intermediate values. Regarding soluble collagen, as a % of total collagen, values were higher (*p* < 0.05) in SM from AL than BI pigs. SM pH_u_ values were not affected by genotype, but significant differences were observed in colour parameters (Table 1). Lightness (L*) was lower and redness (a*) higher (*p* < 0.05) on AL than BI pigs, again with their crosses showing intermediate values. These results affected hue angle (H°) and saturation, respectively lower (*p* < 0.01) and higher (*p* < 0.05) in AL than BI pigs (Table 1).

#### 3.1.2. Adipose Tissue Analyses

Chemical composition of DSF was also affected by genotype (Table 2). Moisture content was lower (*p* < 0.001) in AL than in BI pigs, with RI crosses showing intermediate values. Total lipids, inversely related to moisture content, were higher (*p* < 0.001) in AL and RI crosses than in BI pigs (Table 2).

### 3.2. Trial 2: Pigs Slaughtered at ~150 kg BW

Pigs were slaughtered at an average age of 353.6 ± 2.6 days.

#### 3.2.1. Muscle Tissues Analyses

Physicochemical data from SM samples were less affected by genotype in the fattening period. IMF was higher in AL and BI × AL than in BI pigs, but this difference did not attain statistical significance (Table 3). Total collagen was lower (*p* < 0.05) in AL pigs and RI crosses than in BI pigs, with soluble collagen (% total collagen) following the opposite trend without attaining statistical difference. SM pH_u_ was higher (*p* < 0.05) in AL than in BI pigs (Table 3). Regarding colour, genotype only tended to affect lightness (L*) (*p* = 0.067) and yellowness (b*) (*p* = 0.059) values, and therefore hue angle (H°) (*p* < 0.05) was lower in AL and AL × BI than in BI pigs (Table 3).

IMF from GM muscle was higher (*p* < 0.01) in AL and BI × AL than in BI and AL × BI pigs, while total collagen was lower (*p* < 0.01) in AL pigs and RI crosses than in BI pigs. However, soluble collagen (% total collagen) and colour parameters of GM were not affected by genotype (Table 4).

#### 3.2.2. Adipose Tissue Analyses

Chemical data from DSF were affected by genotype, with total protein lower (*p* < 0.001) in AL pigs and RI crosses than in BI pigs, and total lipids higher (*p* < 0.05) in AL than in BI and AL × BI pigs (Table 5). Finally, DSF colour parameters were also influenced by genotype. Redness (a*), chroma (C*), and saturation were lower (*p* < 0.05) in AL than in BI pigs, while hue angle (H°) was higher (*p* = 0.05), with RI crosses showing intermediate values.

## 4. Discussion

Sustainability and animal welfare policies are increasingly being adopted by the food industry in response to consumer demands. These changes can help strengthen pork niche markets and broaden the target audience for small farmers practicing outdoor swine production [23]. However, farmers and researchers must find a way to improve productivity and product quality, and scientifically support product differentiation [23,24]. One way to improve the performance of outdoor finishing pigs is through crossbreeding. AL and (mainly) BI genotypes are not sufficiently studied, namely in the case of muscles other than longissimus lumborum. In addition, currently available information was obtained in trials with very different or not even described rearing and feeding conditions, as well as age/slaughter weights, among other aspects. Therefore, additional studies are required to evaluate different production stages, in controlled experimental environments.

In order to evaluate meat and fat quality from AL and BI pigs, as well as their reciprocal crosses reared outdoors and fed ad libitum with commercial diets, animals were slaughtered at ~65 and 150 kg BW.

### 4.1. Trial 1: Pigs Slaughtered at ~65 kg BW

Growth and carcass data from this trial were previously presented and discussed by Martins et al. [14]. Briefly, AL pigs had a shorter carcass length and lower bone cuts weight than BI, while Ribatejano (RI) crosses showed intermediate values. AL pigs also showed low lean and high fat cuts proportions, while in BI pigs lean cut proportions were more important. This agrees with the presence in both genotypes of the LEPR c.1987T allele, usually associated with higher fatness, that is almost fixed in the fatty AL when compared to the leaner BI pig breed (0.98 vs. 0.26 frequencies, respectively) [3]. The lower lean and higher fat cuts of the carcasses from AL pigs led to a 12.1% lower commercial yield and 44.7% higher fat cuts proportion than those observed for BI. These differences were due to changes in untrimmed shoulder (−9.7%), loin (−15.8%) and untrimmed ham (−13.7%), and in belly (+24.4%) and backfat (+89.5%) cuts. Meanwhile, although RI crosses showed overall intermediate values, their fat cuts proportions were not significantly different from those of BI pigs. These differences led to lower lean-to-fat cuts ratio and higher backfat thickness and ZP (“Zwei punkte”) fat depth in AL than in BI pigs [14].

Muscle physicochemical traits were affected by genotype. In SM, IMF content was 25.5% higher in AL than in BI pigs, confirming the precociously high adipogenic activity in the AL pig [4]. Strongly influenced by genotype, IMF is positively correlated with the sensory properties, juiciness and palatability of meat [25,26,27]. Meanwhile, compared to IMF values observed in 100 kg AL pigs fed at 85% ad libitum [20,28], and BI pigs [29], those observed in our trial were slightly higher in AL and similar in BI. The differences in the AL breed results, were probably due to different feeding conditions used in both trials.

Myoglobin content has been suggested as a genotype-related characteristic [30]. In our trial, myoglobin showed higher values in the SM muscle from AL pigs and RI crosses when compared to BI pigs. On the other hand, SM total and soluble collagen were respectively 17.4% lower and 25% higher in AL than BI pigs, with RI crosses showing intermediate values. Collagen proteins are the predominant constituents of skeletal muscle connective tissue network and a contributing factor to meat’s texture [27,31]. Likewise, IMF content affects muscle cut resistance, with higher fat corresponding to lower shear force values [27,32]. Therefore, differences observed in IMF and collagen content of SM suggest a more tender meat in growing AL pigs, and tenderness is described as the most important factor for the perceived sensory quality of pork [33]. This trend was also observed in the longissimus lumborum (LL) samples from these animals [14]. Still, total collagen values were higher in SM than in LL, confirming that hindquarter muscles used for locomotion such as biceps femoris, semimembranosus, and semitendinosus, are inherently tougher than support muscles such as longissimus lumborum [27]. Finally, the pH_u_ values, not affected by genotype, were close to the lower value of the normal range in pork, which varies between 5.5 and 5.8 [34].

Consumer’s critical first impression of meat depends mainly on colour, which is in turn largely associated to myoglobin concentration and its chemical form. Other factors, such as the physical state of meat, including pH value, protein state, denaturation degree, and water loss, are also important [30]. SM values for colour coordinates in the literature are scarce and vary widely among breeds (e.g., 36–57, 3–17, and 4–15, for L*, a*, and b*, respectively) [7,20,29,35,36,37]. Values observed in this trial are within the above-mentioned ranges. In our trial, the SM lowest levels of L* and H° and the highest levels of a* and saturation were observed in AL pigs, indicating a darker and redder meat [27]. This agrees with the previously mentioned higher myoglobin content in this genotype. Thus, when compared to BI, AL pigs showed a more intense red SM muscle, as observed in LL [14], which is a distinctive feature for the consumer [38]. The higher L* values observed in SM samples from BI pigs could be partially associated to a higher muscle water loss in these pigs, already noticed in LL muscle [14]. In fact, the higher amount of muscle free-water provides a more reflective surface for light and is positively correlated to lightness [27,39]. Finally, colour values detected in muscle samples from RI crosses were overall intermediate to those observed in AL and BI genotypes, except for H°, closer to the AL values in BI × AL and to the BI values in AL × BI pigs. The lower H° values observed in BI × AL pigs measure a colour closer to the true red axis and agree with a significantly high content in myoglobin in these pigs. Similar results were previously observed in biceps femoris from Duroc × Iberian pigs, when compared to Iberian × Duroc [40].

DSF chemical composition was also affected by genotype, with AL pigs showing a 33.6% lower moisture and an 18.9% higher total lipids content than BI pigs, confirming precociously high adipogenic activity of AL [4].

### 4.2. Trial 2: Pigs Slaughtered at ~150 kg BW

Growth and carcass data from this trial were presented and discussed by Martins et al. [14]. Briefly, in the fattening period AL pigs had a lighter bone structure and a more compact body than BI, presenting a shorter carcass, and lower bone cuts proportions. Carcass yields, higher in these older and heavier pigs, increased 1.16, 0.80, 1.17, and 0.97 percentage units for each 10 kg increase in BW from 65 to 150 kg in AL, BI, AL × BI, and BI × AL pigs, respectively. This confirms fat deposition as the main responsible for increasing carcass yield in older pigs [41,42]. AL pigs also showed a higher fat cuts proportion and a higher backfat thickness and ZP fat depth than BI, influencing the lean-to-fat cuts ratio, lower in AL pigs when compared to BI, and with RI crosses showing intermediate values.

SM muscle was only affected by genotype in total collagen content and pH_u_ parameters. IMF was 11% higher in AL than in BI pigs, however this difference did not attain statistical difference. When compared to BI, SM muscle samples from AL pigs also showed a higher percentage of IMF at 65 than at 150 kg BW (+23.4 and +11.1%, respectively). This was also observed in LL [14], suggesting that AL is an early maturing breed. Total protein values from AL were comparable to those previously reported for 100 kg castrated AL pigs fed at 85% ad libitum [20,28], but IMF values were higher, probably due to different feeding regimes and slaughter weight. Total protein and IMF values from BI pigs from our trial were identical to the ones reported by Carvalho [29].

Although the myoglobin content was 10.3% higher in SM from AL when compared to BI pigs, this difference did not attain statistical significance, contrary to what was observed in trial 1. Meanwhile, myoglobin content increased in all genotypes between 65 and 150 kg BW (186 and 354 days of age), showing that pork gained a more intense colour with age [27], as previously observed in Iberian pigs [43]. On the other hand, SM total collagen content observed in this trial in free-range AL pigs, was higher than the one in 100 kg confined AL pigs [20]. At 150 kg BW, SM total collagen and soluble collagen were also 25.1% lower and 20.6% higher in AL than in BI pigs, respectively. RI crosses also showed lower total collagen values than BI pigs. These differences suggest a higher tenderness of pork from AL pigs and RI crosses. Furthermore, ageing animals show a higher number of stable bonds between collagen molecules, with the corresponding decrease in its solubility [27,43,44], as observed in our pigs slaughtered at 65 and 150 kg. As animals age, meat becomes tougher, mainly due to an increase in the percentage of heat-insoluble collagen bonds [27]. This is a more important factor in local pig breeds than in industrial genotypes, because the former are slaughtered at physiologically older ages.

Ultimate pH (pH_u_) of meat influences water-holding capacity, colour, tenderness, flavour and shelf life of meat [45] and therefore, is a main quality determinant [45,46]. The pH_u_ values observed in SM were within the normal range for pork [34], but were affected by genotype. As already observed in LL [14], pH_u_ values from SM samples were higher in AL than in BI pigs. This suggests a higher muscle glycogen content in BI pigs, positively correlated to lower pH values [47]. Generally, leaner animals have higher percentages of fast-contracting glycogen-rich type IIb or white fibres [48], with a glycolytic metabolism and higher ATP-ase activity, leading to lower pH_u_ values than those observed in slow-contracting oxidative type Ia or red fibres [46].

Meat colour, the major visual factor affecting meat quality [27], was influenced in a less expressive way by genotype in SM during fattening than during growth, as previously observed in LL [14]. Genotype only affected H°, which was significantly lower in SM samples from AL and AL × BI pigs. SM muscle from AL and AL × BI pigs also tended to show lower levels of L* than BI pigs, but a* values were not significantly different. A lower L* and H°, as observed in AL and AL × BI pigs, is related to a darker and redder meat surface in terms of real colour perception [27]. Both darkness and redness can be enhanced at higher pH values, as observed in the SM muscle from AL pigs. In these conditions, reducing and oxygen-consuming enzymes are decreasing the percentage of myoglobin in the oxygenated form, and light scattering is minimized because hydrated muscle proteins are not releasing free water [39]. Overall, when comparing the two trials, an increase in age/weight led to a reduction of L* and an increase of a* values, generally associated to pork with a darker red colour [27]. This difference in L* values from the SM muscle of AL and BI pigs was higher than two units in both trials, which could affect consumers preferences and influence the decision to purchase [49].

Chemical characteristics of GM muscle were slightly affected by genotype. IMF content was higher in AL and BI × AL pigs than in BI and AL × BI pigs, and was comparable to that previously observed in 103 kg BW Iberian pigs fed at 90% ad libitum [50]. Although the difference in IMF between AL and BI was expected, due to the higher adipogenic activity of the former breed [4], IMF values observed in BI × AL, are interesting. In fact, when analysing the IMF values obtained in this trial, BI × AL pigs showed a fat content in both muscles numerically close to the one from AL pigs, suggesting a maternal effect. Since the technological quality of fresh meat and meat products is mainly determined by the lipid fraction, this higher IMF content in the two valuable ham muscles from BI × AL pigs, is very important. Similar results were obtained when comparing IMF content of biceps femoris from hams of Iberian pigs to those of Duroc boars × Iberian dams and of Iberian boars × Duroc dams [51]. Finally, GM total collagen observed in AL pigs and RI crosses was lower than the one in BI pigs. When associated to higher IMF values, as also observed in SM muscle (and in LL muscle—[14]), this suggests a higher tenderness of pork from AL pigs and RI crosses.

DSF chemical composition also varied among genotypes, with AL pigs showing a 29.5% lower total protein and a 6.2% higher total lipids content than BI. Such changes agree with the more adipogenic profile of AL when compared to the leaner BI pig [4,5]. However, histological studies are needed to clarify if the difference in protein content is related to collagen or fat deposition. The latter could be obtained either through an increased adipose cell number in BI pigs and/or by a cell hypertrophy in AL pigs (fewer cells per gram of subcutaneous tissue). When calculating the fat weight deposited in DSF ((DSF weight × % DSF lipids)/100) at 65 and 150 kg BW, the values were 2.88 and 6.31 kg, respectively. Once again, BI × AL pigs showed a total lipids content similar to that of AL, and higher than the one from BI and AL × BI pigs, which is important from a technological point of view. Finally, regarding DSF colour, AL pigs had a 33% lower a* value than BI, which affected C*, H°, and saturation values. The higher lipid concentration in DSF of AL pigs may have contributed to the dilution of blood vessels in this tissue, leading to lower values of a* and saturation, also observed in 65 kg pigs but without attaining statistical significance. In fact, haemoglobin, the major colour pigment in blood, can also affect tissue colour [27].

## 5. Conclusions

Data obtained at the growing period showed that Alentejano (AL) is a fatty breed, with lower lean and higher fat cut proportions than Bísaro (BI) [14]. SM muscle from AL pigs showed higher IMF, redder colour, and lower total collagen, features that could positively influence the consumer from a visual and/or an eating quality point of view. Ribatejano (RI) reciprocal crosses (AL × BI and BI × AL) showed overall intermediate features between AL and BI genotypes, but higher lean and lower fat cut proportions and backfat thickness than AL. On the other hand, they showed a SM muscle with a myoglobin content and colour characteristics in line with those observed in AL pigs. This suggests a redder and darker meat than the one from BI pigs, at a slaughter weight generally used for meat production. These features were overall similar in both muscles of pigs slaughtered at the end of the fattening period (~150 kg BW). At this slaughter weight, muscles from RI crosses also had a lower total collagen content, suggesting a darker, redder, and more tender meat for fresh and cured products than the one from BI pigs. Therefore, RI crosses have the potential to be sustainably reared outdoors and to produce high quality meat and fermented or dry-cured products. AL × BI, more easily reared in the north of Portugal (BI dams’ homeland), could improve the meat and meat products quality when compared to the ones obtained with pure BI pigs. As to BI × AL, more easily reared in the south (AL dams’ homeland), this cross has better commercial yield and primal cuts proportions, when compared to those obtained from pure AL pigs, without compromising meat and meat products quality. Finally, the production of high quality/certified products to attain better market prices can lead producers to increase animal and productivity numbers and therefore contribute to maintaining or increasing animal biodiversity.

## Figures and Tables

**Table 1:** Chemical composition, pH, and CIE colour values of *Semimembranosus* muscle from Alentejano (AL), Bísaro (BI), AL × BI and BI × AL pigs slaughtered at ~65 kg BW (*n* = 10 for each genotype).

Traits	AL	BI	AL × BI	BI × AL	rSD	*p*-Values
Moisture (g/100 g)	71.8 ^b^	74.0 ^a^	72.2 ^b^	72.8 ^a,b^	1.9	0.049
Total protein (g/100 g)	23.8	22.8	23.3	23.2	1.1	0.272
Total intramuscular fat (g/100 g)	5.9 ^a^	4.7 ^b^	5.3 ^a,b^	5.2 ^a,b^	1.0	0.040
Myoglobin content (mg/g)	0.42 ^a^	0.18 ^b^	0.40 ^a^	0.33 ^a^	0.13	0.002
Total collagen (mg/g DM)	15.7 ^b^	19.0 ^a^	17.2 ^a,b^	17.7 ^a,b^	1.9	0.010
Soluble collagen (% total collagen)	11.5 ^a^	9.2 ^b^	10.0 ^a,b^	10.0 ^a,b^	2.1	0.044
pH (24 h *post-mortem*)	5.42	5.52	5.48	5.50	0.14	0.468
Lightness (Cie L*)	43.0 ^b^	46.6 ^a^	45.1 ^a,b^	44.8 ^a,b^	2.9	0.043
Redness (Cie a*)	13.5 ^a^	11.7 ^b^	12.1 ^a,b^	13.1 ^a,b^	1.5	0.049
Yellowness (Cie b*)	6.6	7.1	6.8	7.0	0.8	0.568
Chroma (C*)	15.1	13.8	13.9	14.8	1.6	0.214
Hue angle (Hº)	26.3 ^c^	31.4 ^a^	29.5 ^a,b^	28.3 ^b,c^	2.8	0.004
Saturation	0.36 ^a^	0.30 ^b^	0.31 ^a,b^	0.33 ^a,b^	0.05	0.046

CIE—Commission Internationale de l’Éclairage. AL × BI and BI × AL represent the reciprocal crosses of the commonly known Ribatejano pig. DM—Dry matter. ^a,b,c^ Values in the same row with different superscript letters are significantly different (*p* < 0.05).

**Table 2:** Chemical composition, and CIE colour values of dorsal subcutaneous fat from Alentejano (AL), Bísaro (BI), AL × BI and BI × AL pigs slaughtered at ~65 kg BW (*n* = 10 for each genotype).

Traits	AL	BI	AL × BI	BI × AL	rSD	*p*-Values
Moisture (g/100 g)	7.3 ^c^	11.0 ^a^	8.5 ^b,c^	9.0 ^b^	1.4	<0.001
Total protein (g/100 g)	1.42	1.59	1.42	1.48	0.32	0.811
Total lipids (g/100 g)	85.0 ^a^	71.5 ^b^	81.4 ^a^	81.6 ^a^	4.6	<0.001
Lightness (Cie L*)	82.3	80.7	81.1	80.6	2.5	0.459
Redness (Cie a*)	2.91	3.50	3.18	3.29	0.6	0.257
Yellowness (Cie b*)	4.84	5.17	4.92	4.97	1.1	0.937
Chroma (C*)	5.7	6.2	5.9	6.0	1.2	0.788
Hue angle (H°)	58.3	55.9	56.7	56.4	4.3	0.551
Saturation	0.07	0.08	0.07	0.07	0.01	0.590

AL × BI and BI × AL represent the reciprocal crosses of the commonly known Ribatejano pig. ^a,b,c^ Values in the same row with different superscript letters are significantly different (*p* < 0.05).

**Table 3:** Chemical composition, pH, and CIE colour values of *Semimembranosus* muscle from Alentejano (AL), Bísaro (BI), AL × BI and BI × AL pigs slaughtered at ~150 kg BW (*n* = 9 for each genotype).

Traits	AL	BI	AL × BI	BI × AL	rSD	*p*-Values
Moisture (g/100 g)	73.7	73.7	73.6	73.5	0.9	0.968
Total protein (g/100 g)	22.4	22.8	22.7	22.3	0.7	0.410
Total intramuscular fat (g/100 g)	5.0	4.5	4.6	5.1	0.7	0.326
Myoglobin content (mg/g)	1.93	1.75	1.82	1.80	0.36	0.786
Total collagen (mg/g DM)	15.5 ^b^	20.7 ^a^	17.1 ^b^	17.0 ^b^	3.5	0.029
Soluble collagen (% total collagen)	8.2	6.8	7.4	7.8	2.1	0.518
pH (24 h *post-mortem*)	5.76 ^a^	5.51 ^b^	5.67 ^a,b^	5.66 ^a,b^	0.16	0.027
pH fall (45min to 24 h)	0.55	0.65	0.65	0.56	0.29	0.833
Lightness (Cie L*)	35.4	38.4	35.0	35.6	2.7	0.067
Redness (Cie a*)	14.5	14.3	14.0	15.0	1.3	0.434
Yellowness (Cie b*)	6.7	8.0	6.5	8.0	1.4	0.059
Chroma (C*)	16.0	16.5	15.5	17.0	1.6	0.241
Hue angle (H°)	24.6 ^b^	29.1 ^a^	24.5 ^b^	27.8 ^a,b^	3.8	0.039
Saturation	0.46	0.43	0.44	0.48	0.04	0.102

AL × BI and BI × AL represent the reciprocal crosses of the commonly known Ribatejano pig. ^a,b^ Values in the same row with different superscript letters are significantly different (*p* < 0.05).

**Table 4:** Chemical composition, pH, and CIE colour values of *Gluteus medius* muscle from Alentejano (AL), Bísaro (BI), AL × BI and BI × AL pigs slaughtered at ~150 kg BW (*n* = 9 for each genotype).

Traits	AL	BI	AL × BI	BI × AL	rSD	*p*-Values
Moisture (g/100 g)	69.9	70.6	70.2	69.9	1.2	0.529
Total protein (g/100 g)	21.7	22.4	22.2	21.8	1.3	0.580
Total intramuscular fat (g/100 g)	9.0 ^a^	6.2 ^b^	7.0 ^b^	8.7 ^a^	1.6	0.002
Myoglobin content (mg/g)	1.63	1.34	1.38	1.56	0.32	0.205
Total collagen (mg/g DM)	15.2 ^b^	17.9 ^a^	15.7 ^b^	15.3 ^b^	1.4	0.002
Soluble collagen (% total collagen)	8.5	8.8	8.7	8.6	1.6	0.982
pH (24 h *post-mortem*)	5.62	5.58	5.65	5.61	0.14	0.748
pH fall (45min to 24 h)	0.95	0.89	0.72	0.87	0.28	0.274
Lightness (Cie L*)	39.2	40.3	38.7	39.5	2.9	0.702
Redness (Cie a*)	12.3	12.2	11.8	13.0	1.5	0.344
Yellowness (Cie b*)	5.5	5.8	5.4	6.5	1.4	0.387
Chroma (C*)	13.4	13.6	13.0	14.6	1.8	0.333
Hue angle (H°)	23.9	25.1	24.3	25.9	3.4	0.807
Saturation	0.34	0.33	0.33	0.36	0.04	0.329

AL × BI and BI × AL represent the reciprocal crosses of the commonly known Ribatejano pig. ^a,b^ Values in the same row with different superscript letters are significantly different (*p* < 0.05).

**Table 5:** Chemical composition and CIE colour values of dorsal subcutaneous fat from Alentejano (AL), Bísaro (BI), AL × BI and BI × AL pigs slaughtered at ~150 kg BW (*n* = 9 for each genotype).

Traits	AL	BI	AL × BI	BI × AL	rSD	*p*-Values
Moisture (g/100 g)	5.1	5.8	5.6	5.4	0.9	0.519
Total protein (g/100 g)	0.91 ^b^	1.29 ^a^	0.98 ^b^	0.94 ^b^	0.14	<0.001
Total lipids (g/100 g)	88.9 ^a^	83.7 ^c^	85.5 ^b,c^	87.1 ^a,b^	3.3	0.012
Lightness (Cie L*)	79.3	79.1	79.5	78.8	1.1	0.495
Redness (Cie a*)	2.25 ^b^	3.36 ^a^	2.70 ^a,b^	2.70 ^a,b^	0.9	0.042
Yellowness (Cie b*)	4.47	4.91	4.36	4.69	0.6	0.291
Chroma (C*)	5.0 ^b^	6.0 ^a^	5.2 ^a,b^	5.5^ab^	0.9	0.048
Hue angle (H°)	63.3 ^a^	56.4 ^b^	59.0 ^a,b^	60.7 ^a,b^	6.2	0.049
Saturation	0.06 ^b^	0.08 ^a^	0.07 ^a,b^	0.07 ^a,b^	0.01	0.050

AL × BI and BI × AL represent the reciprocal crosses of the commonly known Ribatejano pig. ^a,b,c^ Values in the same row with different superscript letters are significantly different (*p* < 0.05).

## Supplementary Materials

The following are available online at https://www.mdpi.com/2076-2615/10/5/905/s1, Table S1. Chemical composition (g/100 g) of the commercial diets fed to Alentejano (AL), Bísaro (BI), AL × BI and BI × AL pigs slaughtered at ~65 and 150 kg BW.
