# Supplementary material for: Portuguese Local Pig Breeds: Genotype Effects on Meat and Fat Quality Traits
Source: Animals (Basel). 2020 May 22;10(5):905. doi: 10.3390/ani10050905 (PMC7278411; doi:10.3390/ani10050905)
Supplement: Supplementary file 1 [file animals-10-00905-s001.pdf]

animals

MDPI

Supplementary Materials: Portuguese Local Pig Breeds: Genotype Effects on Meat and Fat Quality Traits

Table S1. Chemical composition (g/100g DM) of the commercial diets fed to Alentejano (AL), Bísaro (BI), ALxBI and BIxAL pigs slaughtered at ~65 and 150 kg BW.

	**Diet 1 (28–50 kg BW)**	**Diet 2 (>50–80 kg BW)**	**Diet 3 (>80–150 kg BW)**
Dry matter (DM) (g/100g)	88.4	88.8	88.7
Total ashes	4.44	4.56	4.25
Crude protein (N x 6.25)	15.46	16.64	15.38
Lysine	1.17	1.06	0.95
Methionine + cystine	0.70	0.67	0.62
Tryptophan	0.17	0.20	0.18
Neutral detergent fibre (NDF)	12.67	12.40	12.94
Acid detergent fibre (ADF)	4.47	4.85	5.05
Starch	46.01	44.22	46.22
Total lipids	4.48	5.05	4.73
Palmitic acid (16:0)[1]	0.80	0.75	0.60
Stearic acid (18:0)[1]	0.35	0.32	0.22
Oleic acid (18:1 *n*-9)[1]	1.49	1.43	1.15
Linoleic acid (18:2 *n*-6)[1]	1.48	1.47	1.50
∑ Saturated FA (SFA)[1]	1.23	1.15	0.86
∑ Unsaturated FA (UFA)[1]	3.15	3.08	2.81
Ratio ∑ UFA : ∑ SFA	2.56	2.68	3.27
Digestible energy (MJ/kg)	14.07	14.28	14.12

[1](g/100 g of total fatty acids identified).
